# Transthoracic Echocardiography-guided ECMO Cannulation in the Emergency Department: A Case Report

**DOI:** 10.5811/cpcem.48486

**Published:** 2025-09-23

**Authors:** William Osae, Kevin Gurysh

**Affiliations:** Duke University Hospital, Department of Emergency Medicine, Durham, North Carolina

**Keywords:** extracorporeal cardiopulmonary resuscitation, extracorporeal membrane oxygenation, transthoracic echocardiography, extracorporeal life support, case report

## Abstract

**Introduction:**

Extracorporeal membrane oxygenation (ECMO) is a life-saving intervention that has become more prevalent in the emergency department (ED) for patients with potentially reversible cardiac or pulmonary failure.

**Case Report:**

We report a case of a young male patient who presented in septic shock and ultimately suffered a cardiac arrest in the ED. Extracorporeal membrane oxygenation was initiated after multiple rounds of cardiopulmonary resuscitation proved futile. Transthoracic echocardiography (TTE) was employed in the ED to guide ECMO cannulation, and the patient was able to make a full recovery after a one-month admission in the intensive care unit.

**Conclusion:**

Transesophageal echocardiography and fluoroscopy are often favored over TTE for ECMO cannulation due to greater resolution of the former modalities. Transesophageal echocardiography is invasive, less accessible, and requires greater expertise. Fluoroscopy requires patients to be moved to a catheterization suite and comes with a risk of extra radiation and contrast-induced nephropathy. While the concept of TTE-guided ECMO cannulation is not especially novel, few case reports exist on its emergent deployment in the ED. Here, we discuss a unique case in which TTE proved effective for timely ECMO deployment for a critically ill ED patient.

## INTRODUCTION

Since its first successful use in 1972, extracorporeal life support (ECLS) has become increasingly common on a global scale. When used to circumvent the heart-lung circulation, such as during a coronary artery bypass graft, it is referred to as a cardiopulmonary bypass. When deployed in the emergency department (ED) or intensive care unit (ICU) to facilitate cardiac output, ventilation or oxygenation, ECLS then becomes extracorporeal membrane oxygenation (ECMO). Due to technological advancements over the last few decades, ECMO deployment has expanded from the pediatric population to adults.^1^ A 2015 study by Sauer et al revealed a 400% increase in ECMO use among adults from 2006 to 2011.^1^

Given its efficacy in stabilizing patients with reversible causes of cardiopulmonary failure, the use of ECMO has become more prevalent in EDs and even in the prehospital setting.^2^ When ECMO is used to augment conventional cardiopulmonary resuscitation, it is referred to as extracorporeal cardiopulmonary resuscitation (ECPR). Recent data have shown substantial benefit when ECPR is deployed early in patients with cardiac arrest.^3^

The most common form of ECMO cannulation is dual cannulation such as veno-venous cannulation (VV ECMO) and veno-arterial cannulation (VA ECMO). Veno-venous ECMO primarily provides pulmonary support in patients with respiratory failure such as in acute respiratory distress syndrome. Veno-arterial ECMO introduces a large right-to-left shunt and mainly assists with hemodynamic support. The most common modes of cannulation include transthoracic echocardiography (TTE), transesophageal echocardiography (TEE), and fluoroscopy. Cannulation is typically performed percutaneously, especially in the emergency setting.^4^ Currently, there are no agreed-upon guidelines for venous ECMO cannulation, and there are no studies demonstrating definitively that one method is superior. However, TEE and fluoroscopy are often favored over TTE for ECMO cannulation due to greater resolution of the former modalities.^5^

Transthoracic echocardiography can be used at the bedside to estimate cardiac ejection fraction, assess volume status by looking at the inferior vena cava, determine right ventricular strain, and assess for pericardial effusion. Transthoracic echocardiography provides a fast, noninvasive, and accessible way of locating the guidewire and cannula during ECMO, especially in the hemodynamically unstable patient undergoing ECPR. Previous studies have discussed TEE as a feasible method of ECMO cannulation in the ED, but TEE is more invasive, less accessible, and requires greater expertise.^5^,^6^ Fluoroscopy also has its drawbacks, including the need to move patients to the catheterization suite, the risk of contrast-induced nephropathy, and added radiation. Such a modality would not be ideal in the unstable patient. In this case report, we discuss a unique case in which TTE proved effective for timely ECMO deployment for a critically ill ED patient.

## CASE REPORT

A 24-year-old male patient with no significant past medical history presented to the ED with altered mental status, hypotension, hypoxia, and tachycardia. The ED team began intravenous (IV) fluid resuscitation and prepared for intubation after poor response to high-flow nasal cannula. During setup for intubation, the patient went into pulseless electrical activity and then pulseless ventricular tachycardia necessitating initiation of CPR with administration of IV epinephrine. Intubation was then completed after CPR had begun. Return of spontaneous circulation was achieved after five minutes. However, the patient remained unstable, and intermittently required epinephrine to maintain blood pressure. Broad spectrum antibiotics were administered after blood cultures were obtained.

The ECMO team was contacted and arrived promptly to the ED. Given the urgency of the patient’s clinical situation, the cardiothoracic surgery and ED teams agreed to initiate VV ECMO using TTE for guidance in venous cannulation. Chest radiograph revealed bilateral pulmonary edema. The patient had frothy secretions pooling in the endotracheal tube, and TTE showed a plethoric inferior vena cava (IVC) with a dilated and hypokinetic left ventricle. The aortic outflow tract also appeared narrowed, with echogenic material visualized on the aortic valve suspicious for vegetation.

A phased-array ultrasound probe was placed in the subxiphoid position to identify the IVC emptying into the right atrium. The surgeons at bedside in the ED accessed the bilateral femoral veins after making an open incision and using a seeker needle. Guidewires were subsequently placed through the seeker needles and the tract was serially dilated. The probe was maintained in the subxiphoid position as two guidewires were placed into the bilateral common femoral veins and advanced into the IVC. Ultrasound was used to confirm correct placement of two guidewires close to the caval-atrial junction from the left and right common femoral veins. The image below shows correct placement of two guidewires into the IVC.

*CPC-EM Capsule*

What do we already know about this clinical entity?

*Extracorporeal membrane oxygenation (ECMO) is a life-saving intervention for emergency department (ED) patients with reversible cardiac or pulmonary failure*.

What makes this presentation of disease reportable?

*Transthoracic echocardiography was successfully utilized to facilitate ECMO cannulation in a critically ill ED patient*.

What is the major learning point?

*Transthoracic echocardiography is a safe and fast method for ECMO cannulation in the emergency department*.

How might this improve emergency medicine practice?

*Transthoracic echocardiography is a valuable tool for ED physicians to provide timely care for critically ill patients who need ECMO deployment in the ED*.

After VV ECMO was fully deployed, the patient’s oxygen saturation improved significantly, and he was transferred to the cardiothoracic intensive care unit (ICU). Transesophageal echocardiography performed in the ICU showed that the patient had wide open aortic insufficiency secondary to multiple aortic vegetations concerning for acute endocarditis. He was taken to the operating room for vegetation removal and repair of his aortic valve and root. Although the patient sustained a large-territory unilateral middle cerebral artery infarct, his neurological status improved over the course of his hospitalization with IV antibiotics, supportive care, and physical/occupational therapy. He was discharged home after four weeks.

## DISCUSSION

This case report demonstrates the successful use of TTE for ECMO guidewire placement in a critically ill ED patient. Established modes of guiding ECMO cannulation include TEE, fluoroscopy, and plain radiograph to confirm location of guidewire tip. Transesophageal echocardiography can provide a three-dimensional view of the heart chambers and is especially beneficial when large dual-vessel cannulation is being performed at the level of the superior vena cava. Fair et al discussed TEE as a feasible method of ECMO cannulation during chest compressions in the ED.^7^ However, TEE requires a much higher level of expertise, is less accessible, and is more invasive compared to TTE. Fluoroscopy has also been shown to be an effective way of facilitating ECMO cannulation especially when combined with ultrasound.^8^ Kashiura et al reported lower rates of complications such as cannula malposition and vessel injury when fluoroscopy was combined with ultrasound as opposed to ultrasound alone.^8^ While fluoroscopy offers a safe way to securely dilate the cannula tract and confirm correct cannula flow, it is not without disadvantages, especially in the ECPR setting. Fluoroscopy requires transport to the catheterization suite, a time-consuming process that may not be favorable to the unstable patient. It also comes with an inherent risk of increased radiation and contrast-induced nephropathy, with the latter complication posing greater risk to the ECPR patient with poor end-organ perfusion.

Transthoracic echocardiography provides a timely, less invasive, and accessible method to guide venous cannulation during ECPR without compromising effective chest compression. Transthoracic echocardiography is not only useful for ECMO cannulation but is also reliable in patient selection for ECMO and cardiac activity detection during CPR.^5^ Plain radiography to confirm the tip of the guidewire has been shown to be inferior to echocardiography.^9^ Plain radiography provides a static view and is especially limited in patients with loss of traditional landmarks due to distorted anatomy.^9^

Percutaneous extracorporeal membrane oxygenation has been used for management of persistent shock and cardiac arrest for over three decades.^10^ One meta-analysis showed that survival to hospital discharge was 44.9% of 675 cardiac arrest patients.^11^ Extracorporeal membrane oxygenation not only improves perfusion in such patients but has been shown to be superior in achieving therapeutic hypothermia in post-anoxic encephalopathy patients.^12^ Time to initiation of ECMO is vital in ECPR cases. The average reported time from hospital arrival to ECMO deployment ranges from 19–40 minutes, and the survival rate was about 20% when time from cardiac arrest to ECMO was less than 60 minutes.^13^,^14^

Transthoracic echocardiography has been widely adopted in everyday clinical practice by emergency physicians, hospitalists, and intensivists. Given its ease of accessibility compared to TEE and fluoroscopy, it serves as a powerful tool in real-time guidance for ECMO cannulation in the ED. In this case report, we describe how ECPR in the ED was used to rescue a young patient who had cardiac arrest in the setting of suspected sepsis. Time was of the essence for this patient, and TTE served as a fast and safe method to provide lifesaving care. We acknowledge that TEE is superior for cannulation at the superior vena cava level for visualizing arterial inflow. Nevertheless, in VV ECMO cannulation at the femoral vessel, TTE can be safely used in guiding venous guidewire placement into the IVC using the subxiphoid view.^15^

Transthoracic echocardiography is not without limitations for venous ECMO cannulation. First, the physician’s clinical competency in TTE will primarily determine which imaging modality is best for cannulation. Additionally, depending on the patient’s body habitus and anatomy, TTE may not provide the spatial resolution needed to facilitate ECMO initiation. Furthermore, assessment of the depth of the guidewire into the IVC beyond the right atrium is limited with TTE.^5^ In this case report, TTE was used to identify only the guidewires and not the cannula. Despite these limitations, TTE is a cost-effective and efficient tool in guiding cannulation during ECPR. Further prospective study is needed to elucidate how TTE compares with other techniques for ECMO cannulation in the ED.

## CONCLUSION

Extracorporeal cardiopulmonary resuscitation in the ED has been shown to reduce mortality especially when time to initiation is within 60 minutes. This case report discusses how ECPR guided by point-of-care ultrasound (via transthoracic echocardiography) was used to rescue a young, critically ill patient in the ED. Transesophageal echocardiography and fluoroscopy are often favored over TTE for extracorporeal membrane oxygenation cannulation due to greater resolution of the former modalities. Currently, there are no agreed-upon guidelines for ECMO cannulation, and there are no studies demonstrating definitively that one method is superior. While the concept of TTE-guided ECMO cannulation is not especially novel, few case reports exist on its emergent deployment in the ED. We support the routine use of TTE in guiding timely ECMO cannulation in the ED.

## Figures and Tables

**Image:**
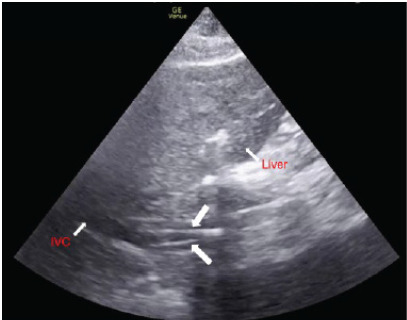
Visualization of two guidewires (large arrows) in the inferior vena cava (IVC) during cannula placement for veno-venous extracorporeal membrane oxygenation.
